# Transcriptomic and chemical analyses to identify candidate genes involved in color variation of sainfoin flowers

**DOI:** 10.1186/s12870-021-02827-8

**Published:** 2021-01-22

**Authors:** Yu Qiao, Qiming Cheng, Yutong Zhang, Wei Yan, Fengyan Yi, Fengling Shi

**Affiliations:** 1 College of Grassland Resources and Environment, Key Laboratory of Forage Cultivation, Processing and High Efficient Utilization of the Ministry of Agriculture and Key Laboratory of Grassland Resources of the Ministry of Education, Inner Mongolia Agricultural University, Hohhot, 010011 China; 2 Inner Mongolia Academy of Agricultural and Animal Husbandry Sciences, Hohhot, China

**Keywords:** Sainfoin, Flavonoid, Anthocyanin, Transcriptome, DEGs, Flower color

## Abstract

**Background:**

Sainfoin (*Onobrychis viciifolia* Scop) is not only a high-quality legume forage, but also a nectar-producing plant. Therefore, the flower color of sainfoin is an important agronomic trait, but the factors affecting its flower phenotype are still unclear. To gain insights into the regulatory networks associated with metabolic pathways of coloration compounds (flavonoids or anthocyanins) and identify the key genes, we conducted a comprehensive analysis of the phenotype, metabolome and transcriptome of WF and AF of sainfoin.

**Results:**

Delphinidin, petunidin and malvidin derivatives were the main anthocyanin compounds in the AF of sainfoin. These substances were not detected in the WF of sainfoin. The transcriptomes of WF and AF in sainfoin at the S1 and S3 stages were obtained using the Illumina HiSeq4000 platform. Overall, 10,166 (4273 upregulated and 5893 downregulated) and 15,334 (8174 upregulated and 7160 downregulated) DEGs were identified in flowers at S1 and S3 stages, respectively (WF-VS-AF). KEGG pathway annotations showed that 6396 unigenes were annotated to 120 pathways and contained 866 DEGs at S1 stages, and 6396 unigenes were annotated to 131 pathways and included 1546 DEGs at the S3 stage. Nine DEGs belonging to the “flavonoid biosynthesis”and “phenylpropanoid biosynthesis” pathways involved in flower color formation were identified and verified by RT-qPCR analyses. Among these DEGs, *4CL3*, *FLS*, *ANS*, *CHS*, *DFR* and *CHI2* exhibited downregulated expression, and *F3H* exhibited upregulated expression in the WF compared to the AF, resulting in a decrease in anthocyanin synthesis and the formation of WF in sainfoin.

**Conclusions:**

This study is the first to use transcriptome technology to study the mechanism of white flower formation in sainfoin. Our transcriptome data will be a great enrichment of the genetic information for sainfoin. In addition, the data presented herein will provide valuable molecular information for genetic breeding and provide insight into the future study of flower color polymorphisms in sainfoin.

**Supplementary Information:**

The online version contains supplementary material available at 10.1186/s12870-021-02827-8.

## Background

Sainfoin (*Onobrychis viciifolia* Scop) is a perennial herbaceous forage legume [1] that is widely distributed in temperate regions of the northern part of the world [2]. It can be used as hay, pellets, grazing and silage because of its high palatability and nutritious forage properties [2–4]. It is particularly valued for having appropriate condensed tannin content to reduce greenhouse gas emissions by preventing bloating in grazing animals [2, 5, 6]. Studies have found that sainfoin can also be used as an ornamental plant because its flowers form an erect raceme and the flowering period is 2–3 weeks [2]. Some studies have also found that sainfoin can be used as a nectar plant due to its beautiful flower petals and high sugar content [7, 8]. Therefore, studying the flower color of sainfoin is of great significance for the development of multifunctional applications.

Flower color is one of the most important horticultural characteristics of plants in nature [9]. Flower color changes can perform important ecological functions by attracting pollinators and affecting the reproductive success of flowering plants [10] and are crucial to plant evolution [11, 12]. In addition, the color of flowers is directly or indirectly related to the agronomic traits of plants, and classic breeding methods have been widely used to develop varieties with flowers varying in both color and intensity [13, 14]. Flower color is affected by many factors, the most important of which are different kinds of plant pigments, such as flavonoids and anthocyanins [15, 16]. Anthocyanins are part of flavonoids, that are the main components to flower pigments, and they are produced by highly conserved structural and regulatory components [17, 18]. During the flowering process, somatic mutations from recessive white to pigment- reversible alleles occur, and the variegation of flowers is inevitably the result of differential gene expression regulation [19]. The anthocyanin biosynthetic pathway includes multiple metabolic processes involving seven core structural genes, such as *CHS*, *LAR*, *DFR*, and *ANS*, as well as several branching enzyme genes [20]. So far, genes associated with flower color and flavonoids have been found in many plants, such as white clover (*Trifolium repens*), alfalfa (*Medicago sativa*), white *Primula vulgaris*, and *strawberry (Fragaria × ananassa)* [14, 18, 21, 22]. However, the molecular mechanisms of the corresponding candidate genes underlying flower pigmentation in sainfoin are still unclear.

Transcriptome technology can provide unique insights into the molecular characteristics of nonmodel plants without a reference genome, especially in the study of flower color. It has been successful in many plants, for example, sheepgrass (*Leymus chinensis*), Siberian wildrye (*Elymus sibiricus*), ornamental crabapple (*Malus prunifolia*) and chrysanthemum (*Dendranthema morifolium*) [23–26]. As far as we know, there has no report of research employing RNA-Seq to study the color of sainfoin flowers. Therefore, the mechanism of color mutation in sainfoin should be understood, and the key genes should be identified. In our study, WF of a sainfoin mutant resulting from EMS treatment and AF were used as the experimental model. Transcriptome technology, CIELAB color space and UPLC were used to assess the variation in related genes and the differences in flavonoid intermediates in the anthocyanin biosynthetic pathway that cause color transitions. The results of this study provide a theoretical basis for future sainfoin molecular breeding, provide an important molecular basis for further studies on colored-flower sainfoin and are crucial for understanding the color formation mechanism.

## Results

### Petal color measurements

The petal color parameters of sainfoin are shown in Table 1. The L* value, which varies in color scale from 0 (black) to 100 (white) and represents lightness, in our study was 28.67 in WF and 21.02 in AF. The a* value, which represents redness, was 3.58 in WF and 13.59 in AF. The b* value, which represents blueness, was 10.75 in WF and 2.32 in AF. The C* value, which represents color vividness, was 11.29 in WF and 13.79 in AF. The h° value, which represents basic color, was 71.58 in WF and 9.70 in AF. In summary, WF had higher (*P* < 0.05) L*, b* and h^°^ values than AF. In contrast, the a* and C* values of WF were significantly lower than those of AF (*P* < 0.05). Therefore, these factors indicate that the petal colors of sainfoin are different.

**Table 1:** Chromatographic parameters of sainfoin petals

Materials	CIELAB coordinate				
	L*	a*	b*	C*	h°
WF	28.67 ± 0.44a	3.58 ± 0.26b	10.75 ± 0.23a	11.29 ± 0.35b	71.58 ± 1.71a
AF	21.02 ± 0.27b	13.59 ± 0.42a	2.32 ± 0.06b	13.79 ± 0.58a	9.70 ± 0.61b

L* means lightness, a* and b* means chromatic components, C* means chroma, h^°^ means hue angle. Values with different letters in the same column are significantly different (*P* < 0.05).

### Major classes of coloration compounds in sainfoin petals

UPLC analysis revealed that seven flavonoids and their derivatives were detected in both the WF and AF: kaempferol-3-O-rhamnosylrutinoside, rutin, kaempferol-3-O-glucoside-phenylpropanoic ester, quercetin-3-O-glucoside, kaempferol-3-O-rutinoside, isorhamnetin--3-O-rutinoside and kaempferol-3-O-glucoside (Table 2). However, three flavonoids (kaempferol-3-O-glucoside-p-courmaric ester, quercetin-3-O-rhamnosidel-p-courmaric ester, kaempferol) were detected in AF but not WF. Among these compounds, only kaempferol-3-O-glucoside-phenylpropanoic ester showed a higher content in WF than AF (*P* < 0.05), and the other flavonoid and derivative contents were lower in WF than in AF (*P* < 0.05). However, five anthocyanidins and their derivatives (delphinidin-3,5-diglucoside, petunidin-3,5-diglucoside, delphinidin-3-rutinoside, petunidin-rutinoside and malvidin-rutinoside) were only detected in AF. No anthocyanidin was detected in WF. Similarly, two procyanidins were detected in our study; only proanthocyanidin was detected in WF, while catechin hydrate was detected only in AF (Table 2). In summary, the differences in the types and contents of coloration compounds in sainfoin petals were the main reasons for the color change.

**Table 2:** The flavonoid, anthocyanidin and procyanidin contents of petals in sainfoin

Substance		WF (μg/g)	AF (μg/g)
Flavonoids	Kaempferol-3-O-rhamnosylrutinoside	1107.23 ± 113.71b	1511.58 ± 128.24a
	Rutin	295.03 ± 22.35b	741.71 ± 111.61a
	Kaempferol-3-O-glucoside-p-courmaric ester	–	169.33 ± 31.25
	Kaempferol-3-O-glucoside-phenylpropanoic ester	379.73 ± 48.03a	232.45 ± 21.57b
	Quercetin-3-O-glucoside	29.99 ± 2.59b	476.43 ± 38.78a
	Quercetin-3-O-rhamnosidel-p-courmaric ester	–	512.69 ± 42.96
	Kaempferol-3-O-rutinoside	1816.62 ± 56.42b	3499.48 ± 392.30a
	Isorhamnetin-3-O-rutinoside	33.08 ± 2.66b	145.76 ± 24.06a
	Kaempferol-3-O-glucoside	439.76 ± 23.23b	2053.49 ± 63.21a
	Kaempferol	–	12.50 ± 2.38
Anthocyanidins	Delphinidin-3,5-diglucoside	–	584.81 ± 26.80
	Petunidin-3,5-diglucoside	–	3310.82 ± 80.96
	Delphinidin-3-rutinoside	–	8650.70 ± 409.36
	Petunidin-rutinoside	–	918.77 ± 15.99
	Malvidin-rutinoside	–	3121.98 ± 84.71
Procyanidins	(+)- Catechin hydrate	–	61.87 ± 2.98
	Proanthocyanidin	3.30 ± 0.53	–
Total		41,04.74 ± 177.65b	26,004.38 ± 1369.67a

### De novo assembly of the sainfoin transcriptome

Twelve libraries of total RNA extracted from sainfoin petals (WF and AF) at the S1 and S3 stages were constructed for transcriptome sequencing. A total of 547,329,260 raw reads with a total of 82,099,389,000 nt were obtained. Then low-quality reads were filtered out, a total of 546,658,468 clean reads with a total of 81,319,631,904 nt were obtained from the twelve sequencing libraries for further analysis (Additional file 1). Briefly, after removing low-quality and contaminating reads, clean reads were retained for further analysis. Finally, Trinity method was used to assemble a total of 53,009 unigenes with an N50 of 1587 nt, with lengths ranging from 201 to 15,519 nt and a mean length of 903 nt (Additional file 2).

### Gene annotation of the sainfoin transcriptome

**Fig. 1:**
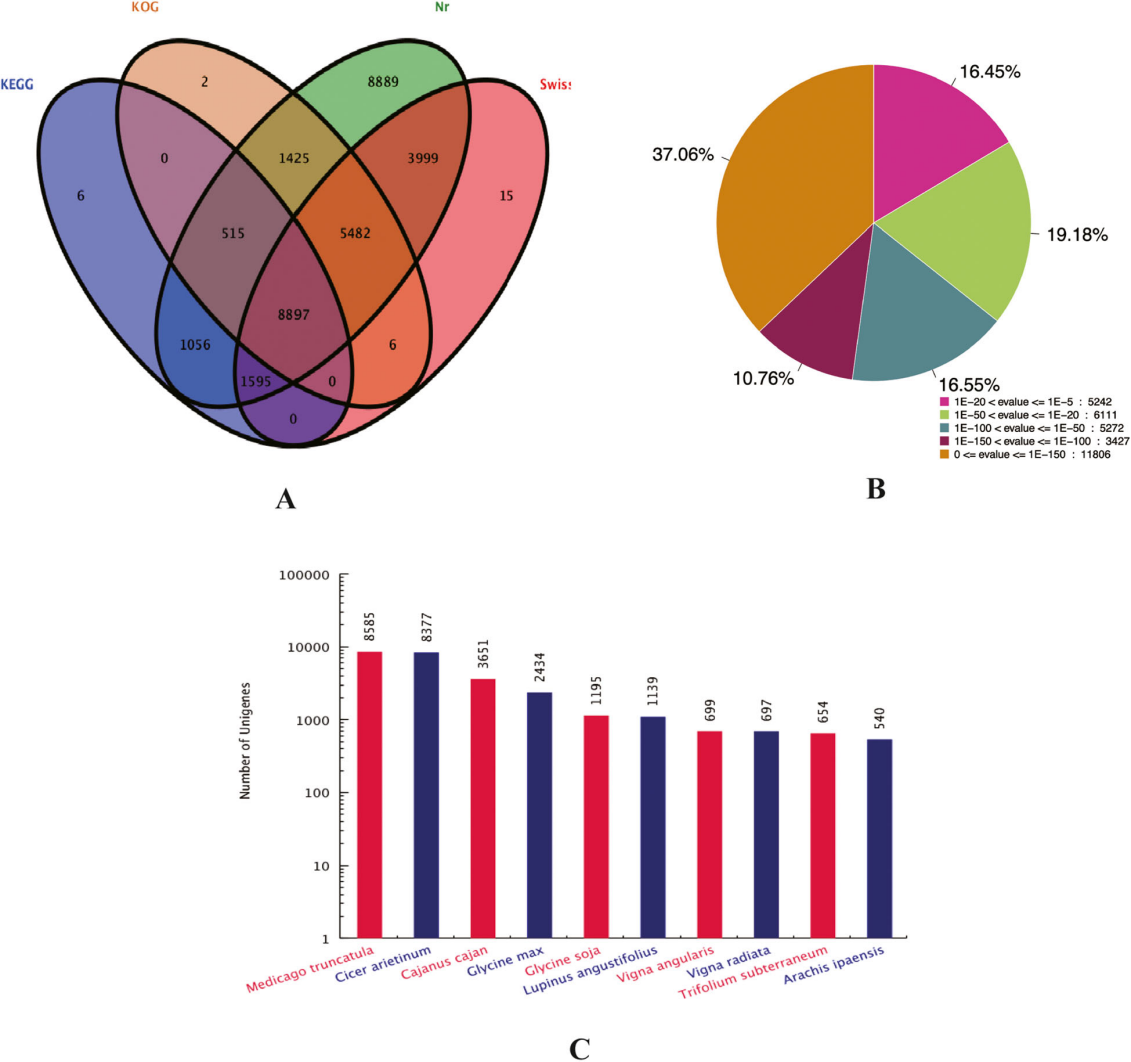
Homology search of sainfoin unigenes. **a**: Venn diagram of sainfoin about number unigenes annotated by BLAXTx. The numbers in the circles indicate the number of unigenes annotated by single or multiple databases. **b**: E-value distribution of the top BLASTx hits against the Nr database. **c**: Number of unigenes in the top 10 species using BLASTx alignment in the Nr database

In total, 31,887 unigenes (60.15% of the 53,009 total unigenes) were annotated against at least one database using BLASTx (E-value < 1 × 10^− 5^) (Table 3). Among 31,858 unigenes (60.10%), 19,994 (37.72%), 16,327 (30.80%) and 12,069 (22.77%) were annotated to the Nr, Swiss-Prot, KOG and KEGG databases, respectively (Table 3). In addition, 6, 2, 15 and 8889 unigenes were annotated to only the KEGG, KOG, Swiss-Pro and Nr databases, respectively (Fig. 1a). According to the Nr database, a total of 5242 unigenes (16.45% of the total 31,858 unigenes annotated to Nr) showed homology (1 × E^− 20^ < E-value ≤1 × E^− 5^), 11,383 (35.73%) unigenes showed strong homology (1 × E^− 100^ < E-value ≤1E^− 20^), and 15,233 (47.82%) unigenes showed very strong homology (E-value ≤1E^− 100^) (Fig. 1b). For the distribution of the species hits obtained by BLAST, 8585 unigenes matched the homologous sequences of *Medicago truncatula*, while 8377, 3651, 2434, 1195 and 1139 unigenes matched the homologous sequences of *Cicer arietinum*, *Cajanus cajan, Glycine max*, *Glycine soja* and *Lupinus angustifolius*, respectively (Fig. 1c). Based on GO analysis, a total of 11,558 (21.80%) unigenes were successfully annotated using GO assignments and categorized into three main categories: biological process, cellular component and molecular function (Additional file 3).

**Table 3:** Assembly of sainfoin Transcriptome

Annotation	Number of unigenes	Annotation percentage (%)
Annotation in Nr	31,858	60.10
Annotation in Swiss-Prot	19,994	37.72
Annotation in KOG	16,327	30.80
Annotation in KEGG	12,069	22.77
Annotation genes	31,887	60.15
without annotation genes	21,122	39.85
Total unigenes	53,009	100.00

### Identification and analysis of DEGs

**Fig. 2:**
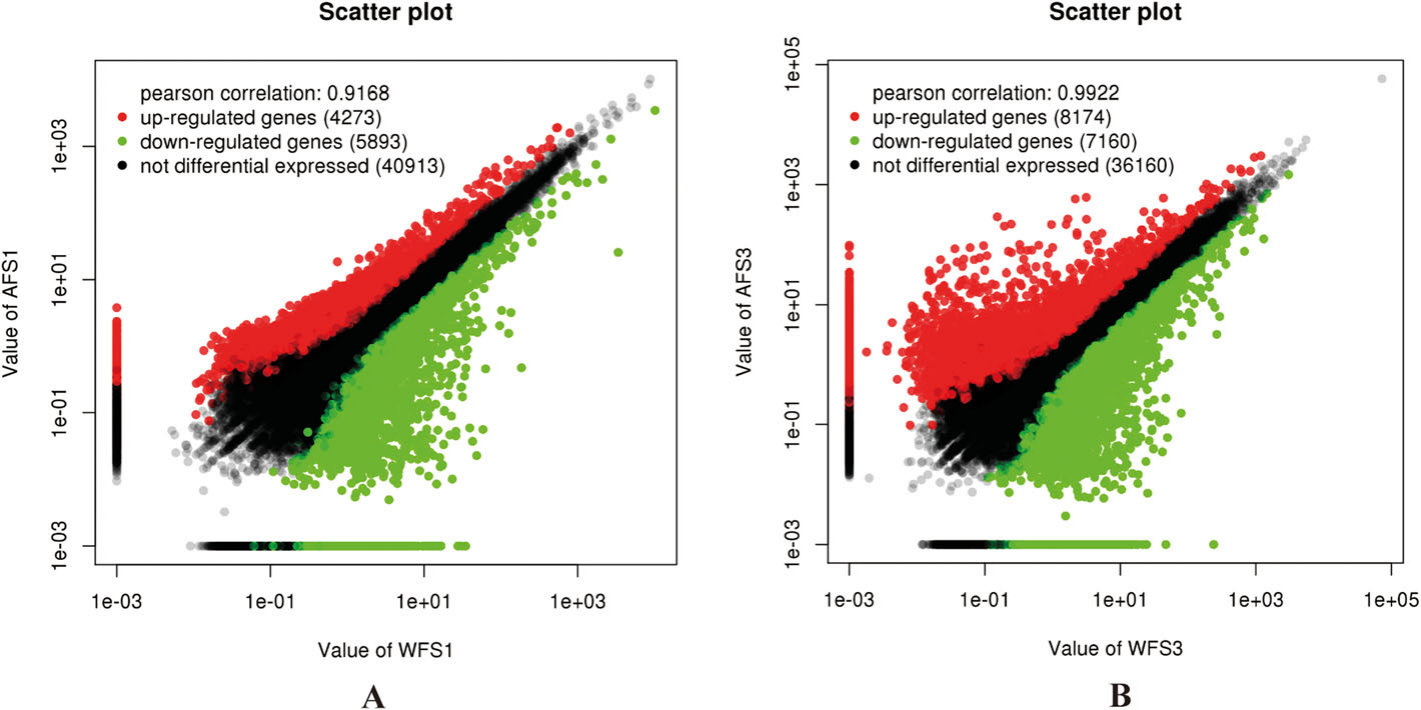
Volcano map of differential gene analysis of sainfoin. **a**: DEGs of WF and AF in S1 stage (WFS1-VS-AFS1). **b**: DEGs of WF and AF in S3 stage (WFS3-VS-AFS3)

Genes acquired by the transcriptome with a false discovery rate (FDR) < 0.05, absolute log2 ratio ≥ 1 were selected as significant DEGs for subsequent analysis (Additional file 4, Fig. 2). To analyze the difference in flower color formation in WF and AF, we compared the DEGs of WF and AF at the same flower developmental stage. Based on these analyses, in the S1 stage, we identified 4273 upregulated DEGs and 5893 downregulated DEGs (WFS1-VS-AFS1) (Fig. 2a). Similarly, in the S3 stage, 8174 unigenes were upregulated, the other 7160 unigenes were downregulated (WFS3-VS-AFS3) (Fig. 2b). The number of DEGs in the S3 stage was greater than that in the S1 stage, indicating that with the prolongation of the growth period, the differences between the WF and AF of sainfoin increased gradually.

### GO analysis of DEGs

To analyze the functions of DEGs, we subjected the DEGs between WF and AF at the same flower developmental stage to enrichment analysis with GO annotation terms. In the S1 stage, a total of 6389 DEGs were divided into three ontologies: biological process, cellular component, and molecular function. For the biological process ontology, “metabolic process”, “cellular process” and “single-organism process” were the most frequent terms and were associated with 801, 684 and 557 DEGs, respectively. For “metabolic process”, there were 376 upregulated unigenes and 425 downregulated unigenes; for “cellular process”, there were 313 upregulated unigenes and 381 downregulated unigenes; for “single-organism process”, there were 269 upregulated unigenes and 288 downregulated unigenes; and “detoxification” (1 unigene), “biological adhesion” (2 unigenes) and “growth” (5 unigenes) were infrequent. For the cellular component ontology, the DEGs were mainly enriched for “cell part” (155 upregulated unigenes, 165 down-regulated unigenes), “cell” (155 upregulated unigenes, 165 downregulated unigenes), “membrane” (131 upregulated unigenes, 150 downregulated unigenes) and “organelle” (109 upregulated unigenes, 135 downregulated unigenes). For the molecular function ontology, the DEGs were mainly associated with the “catalytic activity” (293 upregulated unigenes, 431 downregulated unigenes) and “binding” (285 upregulated unigenes, 344 downregulated unigenes) subcategories (Additional file 5, Fig. 3a). Similarly, in the S3 stage, in total, 11,880 DEGs were divided into three ontologies. For the biological process ontology, the DEGs were also enriched for genes involved in “metabolic process” (797 upregulated unigenes, 699 downregulated unigenes), “cellular process” (704 upregulated unigenes, 600 downregulated unigenes) and “single-organism process” (539 upregulated unigenes, 450 downregulated unigenes). In the cellular component ontology, the DEGs were mainly associated with the “cell part” (384 upregulated unigenes, 261 downregulated unigenes), “cell” (384 upregulated unigenes, 261 downregulated unigenes) and “membrane” (257 upregulated unigenes, 220 downregulated unigenes) subcategories. For the molecular function ontology, the DEGs were also mainly enriched for “catalytic activity” (794 upregulated unigenes, 716 downregulated unigenes) and “binding” (607 upregulated unigenes, 514 downregulated unigenes) (Additional file 5, Fig. 3b).

**Fig. 3:**
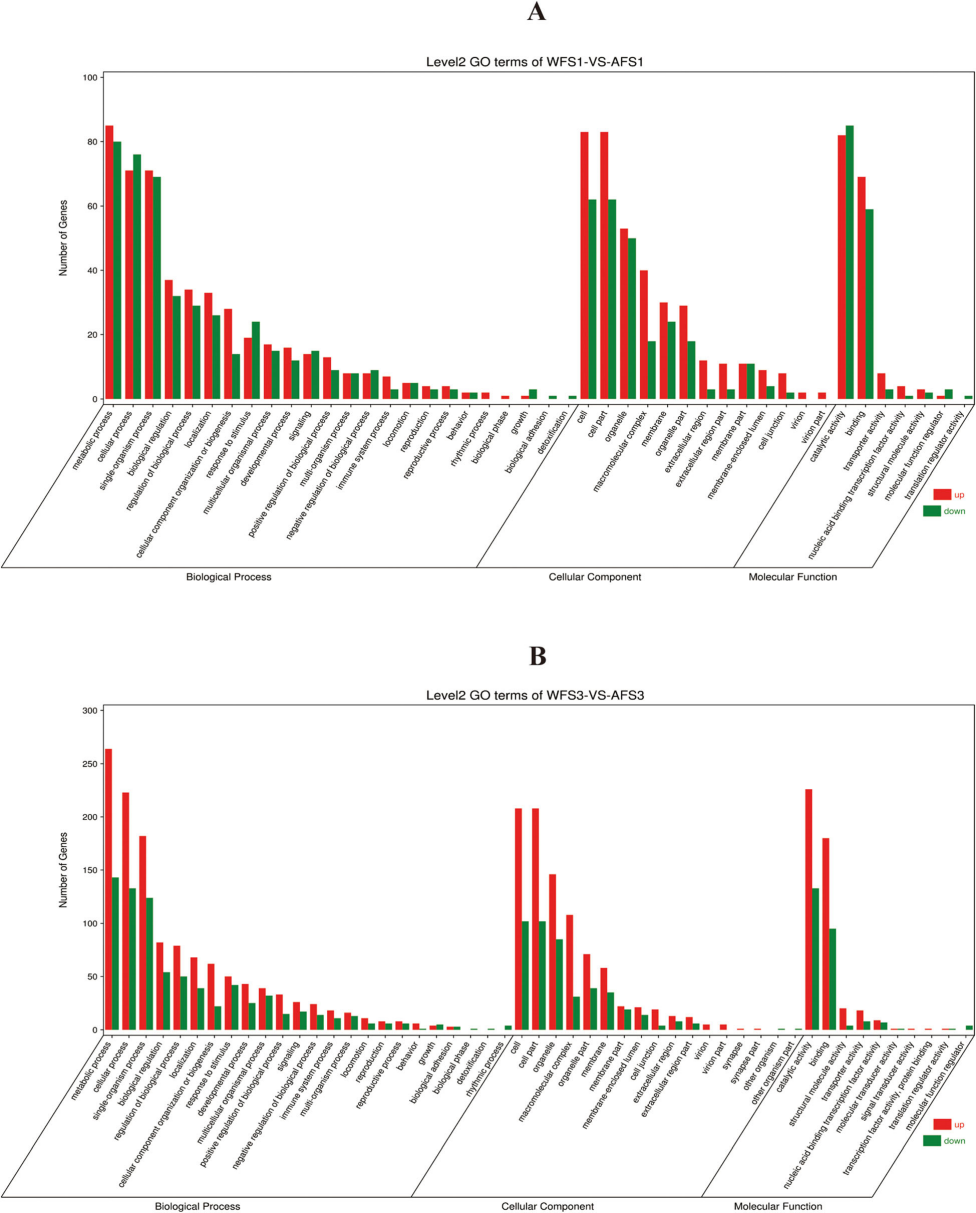
GO classification of DEGs specifically expressed between WF and AF. **a**: GO analysis of DEGs between WF and AF in the S1 stage (WFS1-VS-AFS1). **b**: GO analysis of DEGs between WF and AF in S3 stage (WFS3-VS-AFS3)

### KEGG pathway enrichment analysis of DEGs

To exhaustively explore the biological functions of these DEGs, we carried out an enrichment analysis based on the KEGG database. A total of 6396 unigenes with 866 DEGs were assigned to 120 KEGG pathways in the WFS1-VS-AFS1 comparison and 6396 unigenes with 1546 DEGs were mapped to 131 KEGG pathways in the WFS3-VS-AFS3 comparison (Additional file 6). In the S1 stage, the DEGs between WF and AF were significantly enriched in “flavonoid biosynthesis” (ko00941), “phenylpropanoid biosynthesis” (ko00940) and “biosynthesis of secondary metabolites” (ko01110), and DEGs between WF and AF in the S3 stage were significantly enriched in “biosynthesis of secondary metabolites” (ko01110), “linoleic acid metabolism” (ko00591) and “phenylpropanoid biosynthesis” (ko00940).

In these pathways, the DEGs related to direct or indirect effects on flower color were predicted. Flavonoids, anthocyanins and their derivatives are the main flower color pigments, so we identified a total of three metabolic pathways with eleven genes that control the biosynthesis of flavonoids and anthocyanins (Table 4, Additional file 7). In the “phenylpropanoid biosynthesis” (ko00940) pathway, one DEG (*4CL3*) was annotated. In the “flavonoid biosynthesis” (ko00941) pathway, eight DEGs (*LAR*, *ANR*, *FLS*, *ANS*, *CHS*, *DFR*, *CHI2* and *F3H*) were annotated. In the “flavonoid and flavonol biosynthesis” pathway (ko00944), two DEGs (*FG3* and *PMAT1*) were annotated. All of these genes were used to analyze the expression pattern of the flower color change in sainfoin.

**Table 4:** Partial KEGG pathways associated with flower color of sainfoin

Function	Gene	Enzymes	ID	Enzymes no.
Phenylpropanoid biosynthesis	*4CL3*	4-coumarate: CoA ligase	Unigene0034144	[EC: 6.2.1.12]
Flavonoid biosynthesis	*LAR*	Anthocyanidin reductase	Unigene0025084	[EC: 1.17.1.3]
	*ANR*	Leucoanthocyanidin reductase	Unigene0029546	[EC: 1.3.1.77]
	*FLS*	Flavonol synthase	Unigene0029125	[EC: 1.14.20.6]
	*ANS*	Anthocyanidin synthase	Unigene0014621	[EC: 1.14.20.4]
	*CHS*	Chalcone synthase	Unigene0028334	[EC: 2.3.1.74]
	*DFR*	Dihydroflavonol 4-reductase	Unigene0023972	[EC: 1.1.1.219]
	*CHI2*	Chalcone isomerase	Unigene0035272	[EC: 5.5.1.6]
	*F3H*	Flavanone-3-hydroxylase	Unigene0006821	[EC: 1.14.11.9]
Flavone and flavonol biosynthesis	*FG3*	Flavonol-3-O-glucoside	Unigene0039000	[EC: 2.4.1.239]
	*PMAT1*	Isoflavone 7-O-glucoside-6″-O-malonyltransferase	Unigene0022146	[EC: 2.3.1.115]

### Quantitative real-time PCR analysis of DEGs related to defoliation

**Fig. 4:**
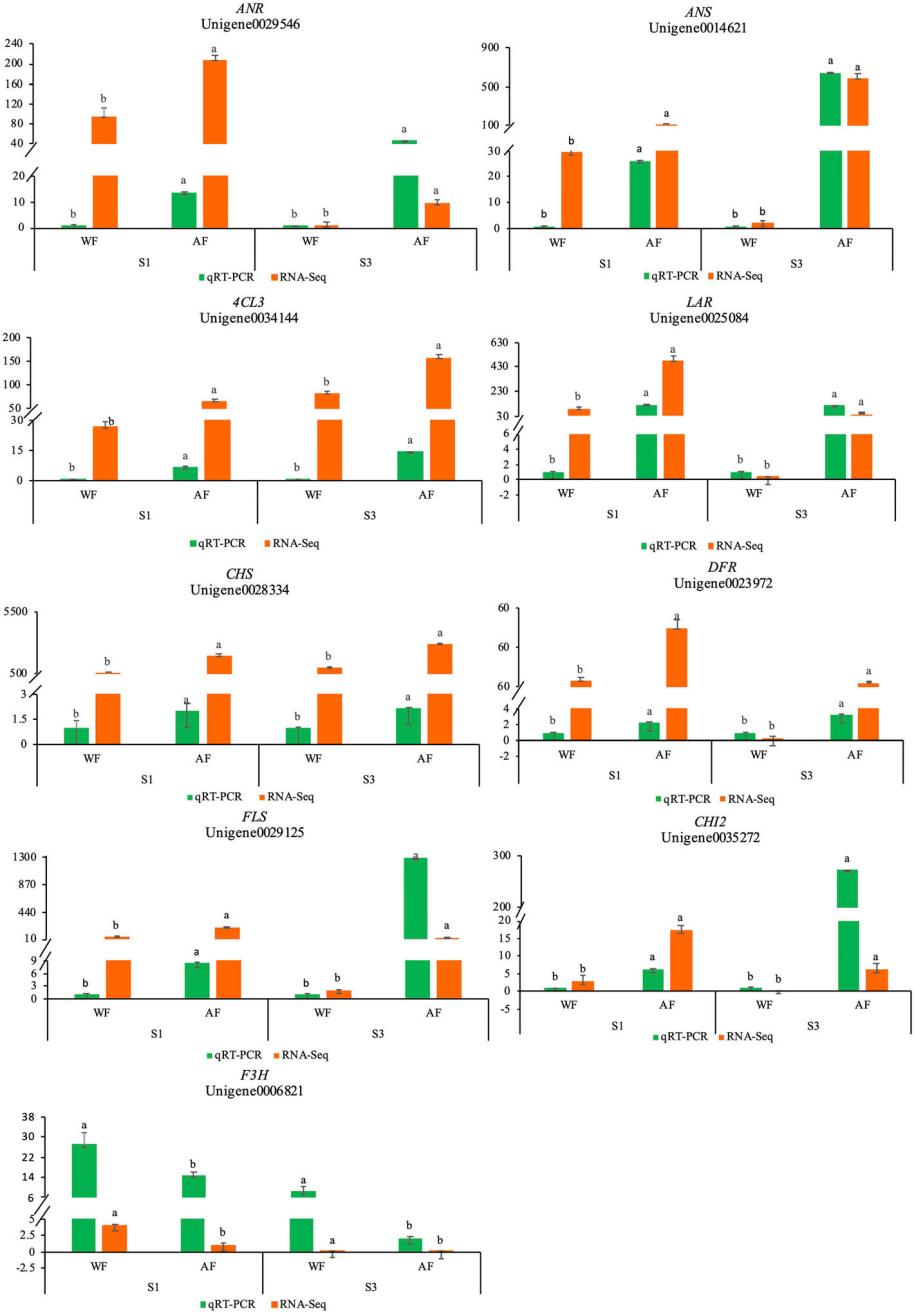
Expression of flower color-related unigenes of sainfoin quantified by RNA-Seq (RNA sequencing) and RT-qPCR (quantitative real-time PCR) analysis. All RT-qPCR reactions were repeated three times for each sample, and vertical bars indicate standard errors

To test the reliability and reproducibility of the RNA-Seq data, gene-specific primers were designed for eleven candidate DEGs. The endogenous reference (Additional file 8) was JZ818469 gene. RNA samples extracted from petals of WF and AF of sainfoin were used as templates, and selected genes related to flower coloration at the S1 and S3 stages were validated based on RT-qPCR. Among the candidate DEGs, only *F3H* had higher expression levels in WF than AF, the other 8 genes of expression levels were all lower in WF (Fig. 4). The significant difference in RT-qPCR data between WF and AF of sainfoin at the S1 and S3 stages was analyzed by t-test, and the results of RT-qPCR exhibited expression patterns almost identical to the RNA-Seq data patterns, which proved the reliability of the RNA-Seq data. In addition, the expression levels of two other genes (*FG3* and *PMAT1*) detected via RT-qPCR were inconsistent with the RNA-Seq data, so we did not show them in Fig. 4.

## Discussion

### Variations in anthocyanin components and color levels between AF and WF

Color mutants are widely used in horticulture and other crops. It has been found that there are many factors leading to flower color mutations in plants, such as ion beam mutations, gamma irradiation and EMS mutagenesis [27, 28]. Among them, flower color mutations induced by EMS have been widely used in cucumber [29], rice [30] and black cumin [31]. However, there is no report on the application of EMS mutagenesis to cause the flower color changes in sainfoin. In this study, we used EMS mutagenesis to obtain WF materials, with AF as control, using HPLC and CLELAB methods to study the chemical substances and phenotypes of AF and WF. At present, the identification and quantitative research on flavonoids and anthocyanins in sainfoin mainly focus on leaves, and less on petals [32, 33]. Regos [34] reported that sainfoin flower buds contained isorhamnetin derivatives, quercetin derivatives, rutin and catechin. In our study, in addition to the above substances, we also detected kaempferol and its derivatives, delphinidin derivatives, petunidin derivatives, malvidin derivatives and proanthocyanidins. In this study, the total flavonoid content of AF was significantly higher than that of WF. Similar results were found in *Primula vulgaris* [35], *Fragaia ananassa* [36] and *Paeonia* [37]. This may be the main reason for the change in flower color in sainfoin. Similarly, we detected delphinidin, petunidin, and malvidin derivatives and catechin in the anthocyanin biosynthetic pathway in only the AF of sainfoin. A similar phenomenon was discovered in the study by Lou [38], who reported that anthocyanins and their derivatives were not detected in the WF of *Muscari armeniacum* f. album. This is because the reduction in anthocyanins causes the petals to lighten in color [39].

Changes in flower phenotype in plants were related to the composition of pigments. Zhong found that the decrease in anthocyanins in *Paeonia lactiflora* resulted in an increase in the L* value and a decrease in the a* value during the flowering period [40]. A similar phenomenon was discovered by Han, who reported that 14 monomolecular anthocyanins in wine were negatively correlated with L*, b* and h° values, and positively correlated with a* and C* values [41]. In our study, we found that the decrease in anthocyanin contents in WF (compared to AF) resulted in an increase in L*, b* and h° values and a decrease in a* and C* values. Our findings are consistent with previous studies. In summary, the change in flower color was closely related to the types and contents of coloration compounds in sainfoin petals, and the synthesis of those compounds was controlled by related genes.

### Genes involved in the flavonoid biosynthesis pathway are differentially regulated

The biosynthesis of flavonoids and anthocyanins has been a research hotspot in the field of plant secondary metabolism, and there is now a good understanding of the nature of related signals and how the signal transduction pathways connect biosynthetic genes [42, 43]. Previous studies have found that flavonoids are one of the most important pigments in many plant petals, and anthocyanins, the end product of the flavonoid biosynthetic pathway, make the widest range of colors, from light yellow to blue-violet [44, 45]. Our results showed that the color difference between AF and WF in sainfoin is due to the loss of malvidin, petunidin, and delphinidin derivatives in WF. This is due to the hindrance of anthocyanin biosynthesis in WF, which is largely regulated by genes [14]. Thus, the key genes for the metabolism leading to WF were identified by comparing the abundances of candidate genes in the AF and WF transcriptomes. We found 9 different genes in 2 metabolic pathways related to flower color formation, namely, the “phenylpropanoid” (*4CL3*) and “flavonoids” (*LAR*, *ANR*, *ANS*, *FLS CH*S, *DFR*, *CHI2* and *F3H*) pathways. In our study, *4CL3*, *LAR*, *ANR*, *FLS*, *ANS*, *CHS*, *DFR* and *CHI2* showed much higher transcription levels in AF than in WF, but *F3H* showed the opposite expression pattern. This indicated that the change in expression of these genes might affect the color change in sainfoin.

The “phenylpropanoid biosynthesis” pathway diverts carbon flow from primary metabolism to secondary phenolic metabolism through the sequential action of *PAL*, *C4H* and *4CL* [46]. *4CL* can transform 4-coumaric acid, erucic acid as well as ferulic acid into homologous coenzyme thiol esters respectively, which is an important step in the biosynthesis of flavonoids and heteroflavonoids [47, 48]. Ehlting [49] cloned the *4CL* gene family in *Arabidopsis thaliana* and demonstrated that *At4CL3* is involved in the biosynthetic pathways of flavonoids. In our study, the RNA-Seq data revealed that sainfoin *4CL* was differentially expressed between AF and WF. Our RT-qPCR results showed that transcription in the AF was approximately 14 times higher than that in the WF. This is the main reason why flavonoid contents in AF are higher than those in WF (Table 2). A similar result was obtained in the study of Duan [14], who reported that the transcriptional expression of *4CL* in purple flowers of alfalfa was higher than that in cream flowers. This indicated that the reduction in *4CL* expression was one of the main reasons for the appearance of white petals in sainfoin.

The “flavanone biosynthesis” pathway is directly related to the biosynthesis and accumulation of flavonoids [50, 51]. The biosynthetic pathway of anthocyanins is a branch of the phenylpropanoid and flavonoid pathways, and anthocyanins are synthesized under the catalysis of a variety of enzymes [52]. Flavanone and anthocyanins play a vital role in flower color formation and diversity in many plants [15]. *CHS* is critical for the production of chalcone, which is the precursor for the synthesis of all anthocyanins and most other flavonoid metabolites [53]. A reduction in *CHS* transcript levels led to WF lines in *Muscari botryoides*, *Petunia hybrida* and *Parrya nudicaulis* [54–56]. Our results showed that *CHS* gene expression in AF was higher than that in WF, which was a good confirmation of previous study results. *CHI* is a key enzyme involved in flavonoid synthesis, and is also one of the enzymes required in the biosynthesis of flavonoid pigments [57]. Previous studies have shown that the decreased expression or insufficient activity of *CHI* will seriously impede the flavonoid biosynthetic pathway in many plants, resulting in significant decreases in the contents of anthocyanin and flavonoid [58, 59], while *CHI* overexpression can increase flavonoid content [60]. In our study, we found that the expression of *CHI2* in AF was higher than that in WF. This is the main reason why flavonoid and anthocyanin contents in AF are higher than those in WF. *F3H* catalyzes the hydroxylation of flavonoids which is necessary for anthocyanin biosynthesis [38]. In this study, the RT-qPCR detected expression of *F3H* in WF was higher than that in AF. Studies on alfalfa and carnation have shown that low expression of *F3H* causes a deeper flower color [14, 61]. This is because the cyanidin metabolism branch is effectively limited by *F3H* gene [18]. Interestingly, proanthocyanidin was found in the white petals, suggesting that the white petals were due to the lack of anthocyanin biosynthetic pathway downstream genes. *FLS* is the main enzyme responsible for the formation of quercetin and rutin [21]. As evidenced by the qRT-PCR results, the *FLS* gene expression in the AF was higher than that in the WF. This is the main reason why quercetin and rutin contents in AF is higher than that in WF (Table 2). The *DFR* gene can reduce dihydroflavonols to colorless leucoanthocyanidins; the *ANS* gene can convert the colorless leucoanthocyanidins into colored malvidin, pelargonidin and delphinidin [15]. The expression of *ANS* in the WF was lower than that in the AF and anthocyanins were detected in the WF in our study (Fig. 4, Table 2); therefore, the ANS gene might be the key factor in the inability to accumulate anthocyanins in the WF, which was similar to that of Li [18]. Many studies have reported that low expression of *DFR* hampers pigmentation in *Arabidopsis*, *Dianthus caryophyllus* and *Dendranthema morifolium* [62–64]. Therefore, it can be inferred that the *CHS*, *CHI2*, *DFR*, *ANS*, *FLS* and *F3H* genes are the key factors that lead to failure to accumulate coloration compounds in WF. Our results are consistent with those of previous studies [31, 51, 52].

The specific production pathways of proanthocyanidins include *LAR* and *ANR*, both of which are key enzymes for their synthesis [65]. *LAR* can convert leucoanthocyanidin into catechin [66]. The *ANR* gene is an anthocyanin reductase that can transform anthocyanidins to flavan-3-ols needed for the proanthocyanidins produced in the flavonoid pathway [67]. In our study, catechin was detected in AF but not WF of sainfoin, and proanthocyanidins showed the opposite pattern. In addition, the RNA-Seq data and RT-qPCR revealed that the expression of the *ANR* and *LAR* genes in AF was higher than that in WF. Overall, the high expression of the *LAR* gene in AF compared with WF led to a higher catechin content in AF. This result was similar to that of Wang [68]. However, our result was opposite to that of Xie [69], who reported that the overexpression of *ANR* in *Arabidopsis thaliana* leaves resulted in anthocyanin loss and proanthocyanidin accumulation. Therefore, the role of the *ANR* gene in flower petals in sainfoin needs further study.

In summary, compared with that of AF, the flavonoid biosynthetic pathway of WF was blocked upstream by *4CL3*. Furthermore, the downregulated expression of *4CL3*, *FLS*, *ANS*, *CH*S, *DFR*, and *CHI2* resulted in a decrease in flavonoid and flavone compounds, such as rutin, kaempferol and its derivatives, reducing anthocyanin synthesis. At the same time, the high expression level of *F3H* might disrupt anthocyanins synthesis, leading to the formation of WF.

## Conclusion

The contents and metabolic pathways of flavonoids and anthocyanins in amaranth and white petals in sainfoin were compared by UPLC, RNA-Seq and RT-qPCR. The main anthocyanins in AF in sainfoin were malvidin, petunidin and delphinidin derivatives, but these anthocyanins were not detected in WF. The main reason for the appearance of WF in sainfoin was the differential expression of multiple genes related to flavonoid and anthocyanin biosynthesis, resulting in the differences in the types and contents of flavonoids and anthocyanins. Our RNA-Seq data greatly enrich sainfoin genomic research. Our results will provide valuable molecular information for genetic breeding and provide a reference for the future study of flower color polymorphisms in sainfoin.

## Methods

### Plant material

**Fig. 5:**
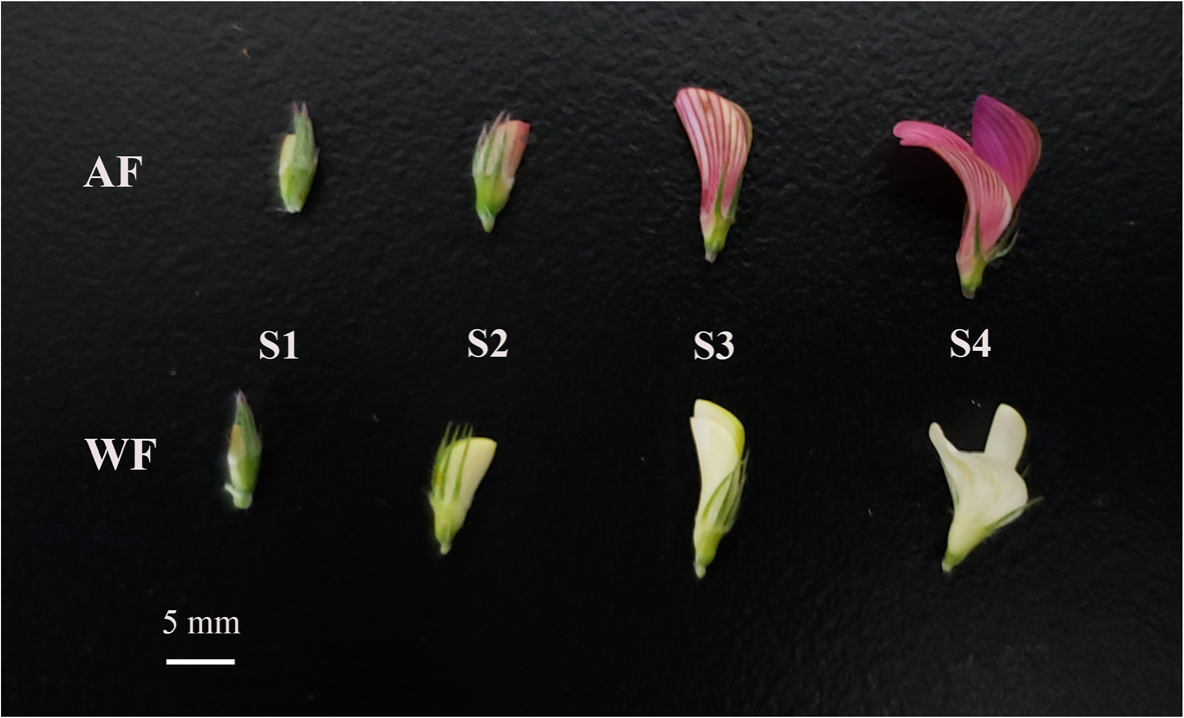
Different flower colors of sainfoin (white flower ‘WF’ and amaranth flower ‘AF’) at four developmental stages

The sainfoin (*Onobrychis viciifolia* Scop ‘Mengnong’) material used in this experiment was provided by Inner Mongolia Agricultural University. This variety was approved by The Chinese Herbage Varietal Resources Registration Board in 1994 and registered as a new variety (Variety registration No.: 151). At the same time, it was also put on record in the Department of Animal Husbandry and Veterinary Medicine, Ministry of Agriculture. The EMS (Sigma Co.) concentration that yielded 50% sainfoin seed lethality (LD50) was 0.9% (v/v) after 18 h. Seeds treated with LD50 were transferred into the field, a WF mutant was found in 2013, and its seeds were collected individually. After mixing and planted for another three generations, WF from F4 generation were confirmed in 2017. In May 2018, AF and WF plants were planted at the experimental base of Inner Mongolia Agricultural University, located in Hohhot, Inner Mongolia, North China (latitude: 40°80’N, longitude: 111°69’E, elevation: 1058 m). The four developmental stages were defined according to the petal changes: S1, calyx higher than petal; S2, calyx and petal equal in height; S3, calyx lower than petal; S4, floret in full bloom (Fig. 5).

### Petal color measurements

Petal color was measured at the S3 stage by a colorimeter (NH300, 3nh, China) with D65 illuminant. The colors are expressed as CIELAB [70] values (L*, a*, b*, C* and h°), and the average of six measurements per flower and ten flowers per treatment was used. L* represents the lightness of the color with a range from black (0 = black) to white (100 = white). The color parameters a* and b* vary from − 60 to 60; a* describes redness and greenness, and b* describes yellowness and blueness. C* is used to denote the saturation of the color, and the higher the C* value was, the more saturated the color was. h° expresses the hue of the color, where 0° = red and 270° = blue [71].

### Extraction and qualitative and quantitative analyses of flavonoids

Freeze-dried petals at stage S3 were ground in liquid nitrogen and powder (20 mg). The 1 mL of 0.1% acetic acid/methanol was used to extract sample at 4 °C overnight. Extracts were centrifuged at 10000 rpm with 10 min. The identification and quantification of flavonoids and anthocyanin compounds were conducted with an ultrahigh-performance liquid chromatograph-mass spectrometer coupled to a triple-quadrupole mass spectrometer (XEVO®-TQ, Waters, Milford, MA, USA) with ESI [34]. The relative anthocyanin and flavonoid contents were computed from the peak areas of the ion peaks of the characteristic Mass spectrometry daughter based on the strength of the corresponding standard compounds. MassLynx™ (V 4.1, SCN 846, Waters Corp., Manchester, UK) was used for Mass spectrometry data acquisition and data analysis. SDs were obtained from three biological replicates.

### RNA extraction, cDNA library construction and sequencing

Petals from the AF and WF of sainfoin at the S1 and S3 stages were sampled, for a total of twelve samples, including three biological replicates. All samples were preserved at − 80 °C for RNA extraction. The total RNA was extracted following the instruction manual of Qiagen RNeasy Plant Mini Kit. The RNA concentration and quality were determined by NanoDrop 2000 (Thermo Fisher Scientific, Waltham, MA, USA). Then the mRNA was enriched after removing rRNA by using Ribo-ZeroTM Magnetic Kit (Epicentre). The cDNA library was built based on enriched mRNA with NEBNext® Ultra™ II RNA Library Prep Kit for Illumina® followed the manufacturer’s instructions. The cDNA was stored at − 80 °C for sequencing and RT-qPCR experiment. The RNA-Seq library was sequenced on an Illumina HiSeq4000 instrument by Gene Denovo Biotechnology Co., Guangzhou, China.

### De novo transcriptome assembly, unigene annotation, and DEG analysis

Our transcriptome datasets were deposited in the NCBI database under a BioProject ID: PRJNA643568. Transcriptome de novo assembly was performed with clean reads filtered from the raw reads by removing adapters, unknown nucleotides (> 10%), and low-quality reads (Q-values ≤10). Then, FastQC (http://www.bioinformatics.babraham.ac.uk/projects/fastqc/) was used to verify sequence quality, including the Q20, Q30 and GC content of clean reads. Since sainfoin (*O. viciifolia* Scop) genome information was not available, the clean reads of all twelve samples were combined for de novo assembly of the transcriptome using the reference genome independent Trinity method [72]. Trinity software package was used to combine the Inchworm, Chrysalis and Butterfly components [73]. First, short clean reads of a certain length were combined with overlap to form longer contigs by inchworm. Second, based on their paired-end information, clean reads were mapped back to the corresponding contigs by Chrysalis. At last, the path that were taken by reads and pairs of reads were analyzed by Butterfly. The finished sequences of the transcripts were defined as unigenes. BLASTx program were used to annotate all assembled unigenes (http://www.ncbi.nlm.nih.gov/BLAST/) with a threshold of E-value < 0.00001 to the Nr database (http://www.ncbi.nlm.nih.gov), Swiss-Prot protein database (http://www.expasy.ch/sprot/), KEGG database (http://www.genome.jp/kegg), KOG database and GO (http://www.geneontology.org) [74]. Unigene expression was normalized to RPKM values and differentially expressed genes were identified among samples or groups by edgeR software with a criteria of |fold change| ≥ 2, and FDR < 0.05 (http://www.r-project.org/). Next, GO and KEGG enrichment analyses were carried out for all DEGs, and hypergeometric tests with *p* ≤ 0.05 as a threshold were used to determine the significant enrichment of GO terms and KEGG pathways.

### RT-qPCR analysis

Eleven selected DEGs involved in flavonoid synthesis were determined by one-step RT-qPCR. The experiment was performed on an ABI 7500 system (Applied Biosystems, USA) using SYBR (TaKaRa). The primers for the DEGs were designed by Primer Premier 5.0 (Premier, Canada) and the reference gene was JZ818469 (Additional file 8) [75]. The relative expression levels of DEGs were analyzed using the 2 ^-△△CT^ method [76]. Each sample (including three biological replicates) was quantified in triplicate.

All the data were subjected to statistical analysis using the t-test (SPSS ver.19.0), and they are presented as the mean ± SD. The effect was considered significant when *P* < 0.05.

## Supplementary Information

**Additional file 1.** Assembly of sainfoin transcriptome.

**Additional file 2.** The size distribution of sainfoin unigenes.

**Additional file 3.** GO classification of assembled unigenes.

**Additional file 4.** DEGs generated from WF and AF of sainfoin in S1 and S3 stages.

**Additional file 5.** GO functional annotations and the number of DEG statistics.

**Additional file 6.** KEGG pathway annotation of DEGs between WF and AF of sainfoin in S1 and S3 stages.

**Additional file 7.** KEGG pathway showing phenylpropanoid biosynthesis, flavonoid biosynthesis, flavone and flavonol biosynthesis in sainfoin.

**Additional file 8.** Primers used for RT-qPCR analysis.

## Data Availability

All raw sequence data are available at NCBI project PRJNA643568 and Sequence Read Archive (SRA) with accession number SRR12130587, SRR12130586, SRR12130585, SRR12130584, SRR12130583, SRR12130582, SRR12130581, SRR12130580, SRR12130579, SRR12130578, SRR12130577 and SRR12130576. The addresses are as follows: https://submit.ncbi.nlm.nih.gov/subs/sra.

## Abbreviations

DEGs: Differentially expressed genes
nt: nucleotides
BLAST: Basic Local Alignment Search Tool
Nr: NCBI nonredundant protein
KEGG: Kyoto Encyclopedia of Genes and Genomes
KOG: Clusters of Orthologous Groups of proteins
Swiss-Prot: A manually annotated and reviewed protein sequence database
GO: Gene Ontology
RPKM: Reads per kb per million reads
RT-qPCR: Real-time quantitative PCR
ESI: Electrospray ionization
SDs: Mean values and standard deviations
*4CL*: 4-coumarate: CoA ligase
*ANS*: Anthocyanidin synthase
*CHI*: Chalcone isomerase
*CHS*: Chalcone synthase
*DFR*: Dihydroflavonol 4-reductase
*F3H*: Flavanone-3-hydroxylase
*FLS*: Flavonol synthase
*LAR*: Anthocyanidin reductase
*ANR*: Leucoanthocyanidin reductase
*FG3*: Flavonol-3-O-glucoside
*PMAT1*: Isoflavone 7-O-glucoside-6″-O-malonyltransferase
UPLC: Ultra-high performance liquid chromatography–mass
WF: White flower
AF: Amaranth flowers
