# Supplementary material for: Transcriptomic and chemical analyses to identify candidate genes involved in color variation of sainfoin flowers
Source: BMC Plant Biol. 2021 Jan 22;21:61. doi: 10.1186/s12870-021-02827-8 (PMC7825240; doi:10.1186/s12870-021-02827-8)
Supplement: Supplementary file 2 — Additional file 2. The size distribution of sainfoin unigenes. [file 12870_2021_2827_MOESM2_ESM.docx]

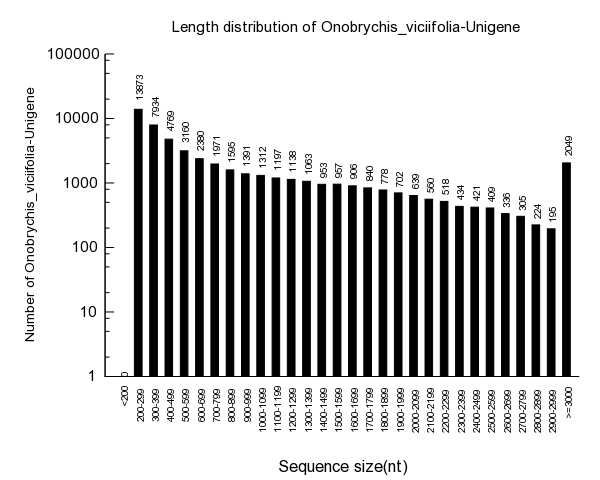


**Fig. S1** The size distribution of sainfoin unigenes (Total: 53,009). The abscissa is the length of the assembled unigenes from 200 nt to ≥ 3,000 nt, and the ordinate is the number of unigenes of the corresponding length.
