# Supplementary material for: Transcriptomic and chemical analyses to identify candidate genes involved in color variation of sainfoin flowers
Source: BMC Plant Biol. 2021 Jan 22;21:61. doi: 10.1186/s12870-021-02827-8 (PMC7825240; doi:10.1186/s12870-021-02827-8)
Supplement: Supplementary file 3 — Additional file 3. GO classification of assembled unigenes. [file 12870_2021_2827_MOESM3_ESM.doc]

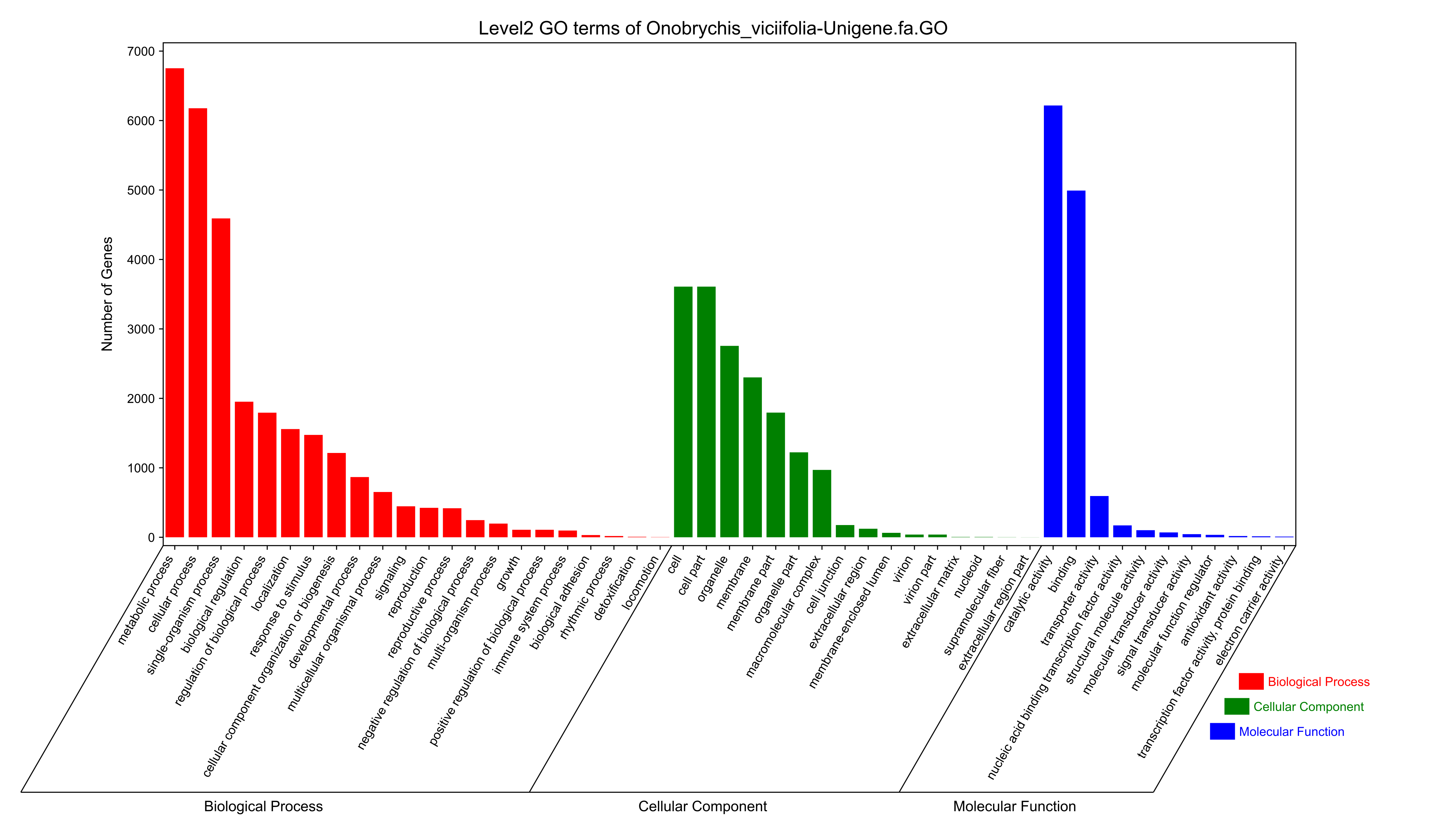


**Fig. S2 GO classification of assembled unigenes.** A total of 11,558 unigenes were categorized into three main categories: biological process, cellular component and molecular function.
