# Supplementary figures and images for: Transcriptomic and chemical analyses to identify candidate genes involved in color variation of sainfoin flowers
Source: BMC Plant Biol. 2021 Jan 22;21:61. doi: 10.1186/s12870-021-02827-8 (PMC7825240; doi:10.1186/s12870-021-02827-8)

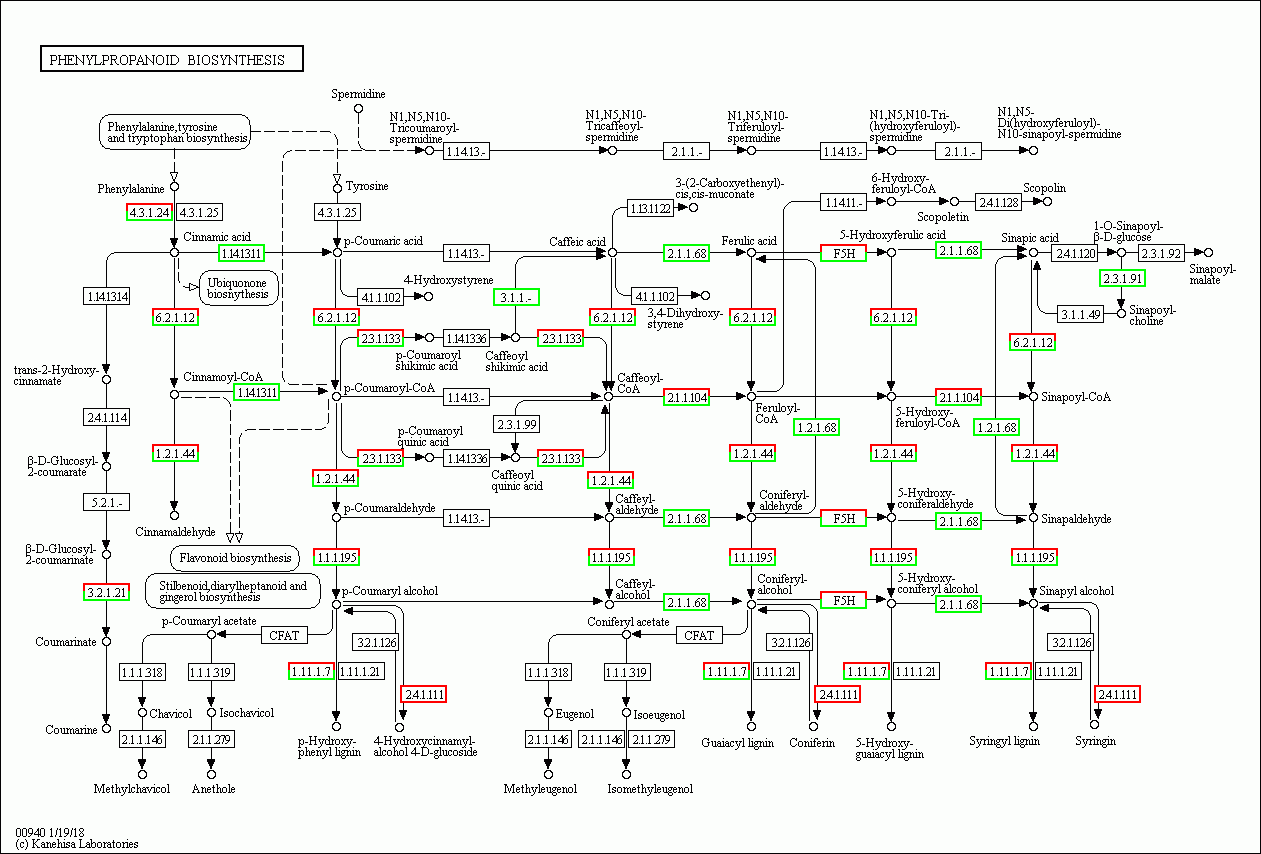


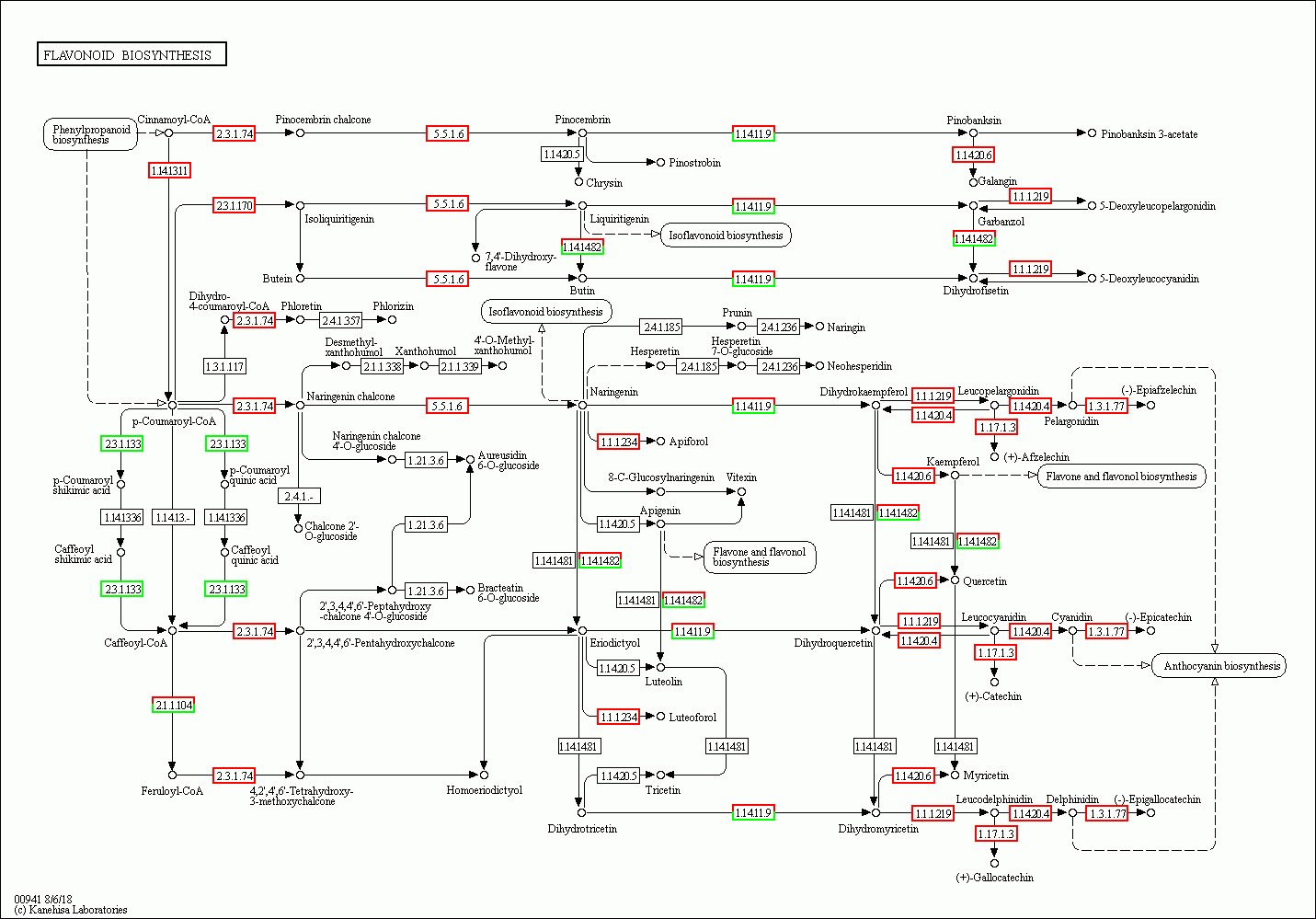


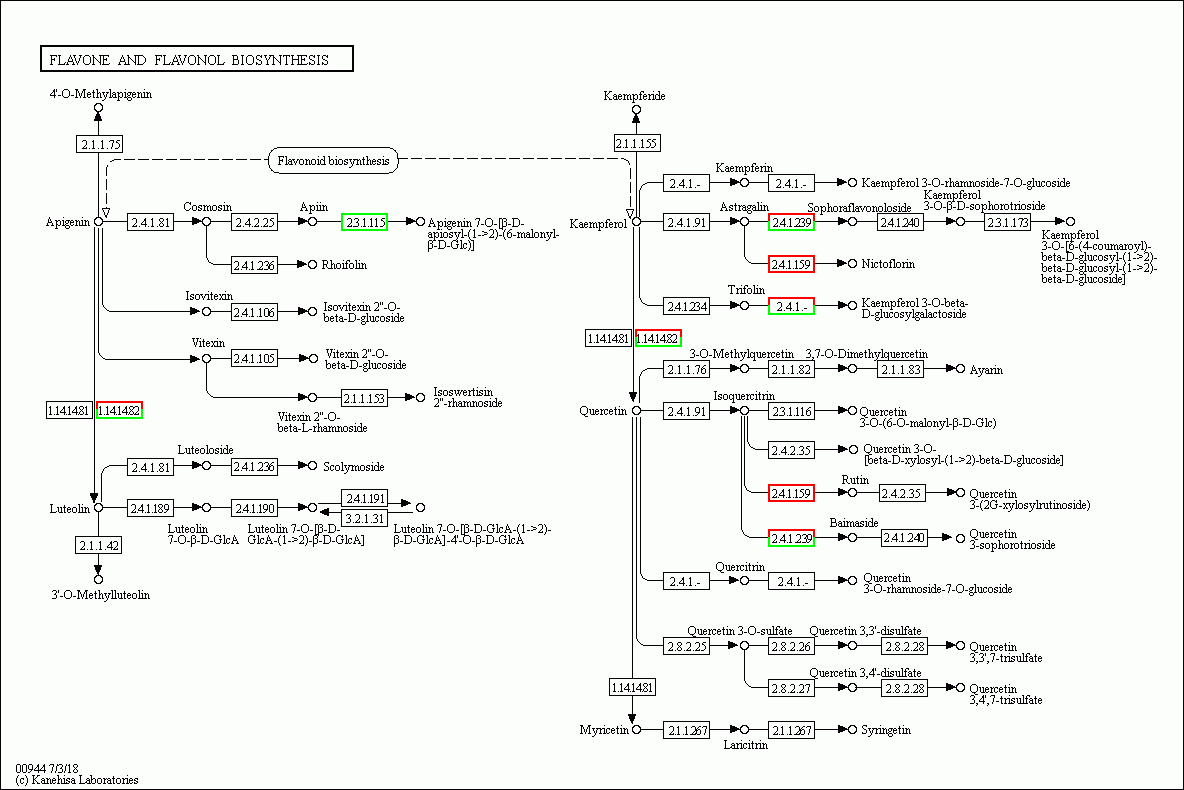

Supplement: Supplementary file 7 — Additional file 7. KEGG pathway showing phenylpropanoid biosynthesis, flavonoid biosynthesis, flavone and flavonol biosynthesis in sainfoin. [file 12870_2021_2827_MOESM7_ESM.docx]
